# New Frontiers and Old Challenges: How to Manage Incidental Findings When Forensic Diagnosis Goes Beyond

**DOI:** 10.3390/diagnostics10090731

**Published:** 2020-09-22

**Authors:** Luciana Caenazzo, Pamela Tozzo, Kris Dierickx

**Affiliations:** 1 Department of Molecular Medicine, Laboratory of Forensic Genetics, University of Padova, 35121 Padova, Italy; luciana.caenazzo@unipd.it; 2 Centre for Biomedical Ethics and Law, Faculty of Medicine—KU Leuven, Kapucijnenvoer 35 Box 7001, 3000 Leuven, Belgium; kris.dierickx@kuleuven.be

**Keywords:** incidental findings, forensics, information disclosure, confidentiality, ethical challenges

## Abstract

Incidental findings (IFs) are well known in medical research and clinical practice as unexpected findings having potential health or reproductive importance for an individual. IFs are discovered under different contexts but do not fall within the aim of a study, and/or are unanticipated or unintentionally revealed, and/or are not the specific focus or target of the particular research or clinical query. Today, in forensic settings, we can consider as incidental findings all the information that is neither related to the cause of death nor to the dynamic of the event or the scope of the forensic investigation. The question whether and how professionals should consider traditional values as guiding notions in the reporting of IFs in the context of forensic assessments is the focus of this article. We propose a descriptive analysis, which focuses on the forensic field, describing forensic situations in which IFs may occur, and whether and to whom they may be disclosed. Some considerations will be provided regarding forensic experts concerning their moral commitment to warn relatives about IFs.

## 1. Introduction

Incidental findings (IFs) have been defined as unexpected findings having potential health or reproductive importance for an individual that are discovered in medical practice under different contexts [1]. IFs provide results outside the scope of the investigation or assessment and providing new information that was not expected. In particular, in clinical practice they have been reported frequently in the contexts of neuroimaging, oncology and genetics. There is a great deal of literature on the ethical reasons presented in the argument-based literature for and against the disclosure of IFs arising in clinical and research genetics contexts, considering different positions as to the patient’s consent regarding the receipt of unexpected information [2,3].

As the new technologies of next generation sequencing pass from being applied in research to being used in the clinic, the large amount of information that these techniques can reveal make IFs a topic of growing and important interest. However, it is easy to understand how some applications of new technologies (for example, Massive Parallel Sequencing) can provide information that is not yet technically nor clinically actionable. In clinical practice, the development of these new technologies can lead to two different situations: genetic information that can provide clinical benefits in terms of prevention, diagnosis and therapy (i.e., medically actionable) can be accidentally discovered, or clinicians may face incidental findings for which clinical manifestations can be uncertain or not yet proven. These incidental findings may be validated and clinically useful, or validated but without any treatment or preventive measures, or have unclear or unknown significance. In the literature, various approaches to the management of incidental/secondary findings in the clinic have been proposed and the debate has focused mainly on discussing which IFs can be identified and which should be revealed and when [4,5,6].

When developing consent procedures that inform patients and/or guardians about this type of result, it is necessary to make a distinction between what is the target of a clinical test or procedure and what is more “off-target”, as well as when devising follow-up procedures and formulating professional obligations. For example, if a physician realizes that a patent is at genetic risk for a potentially fatal, genetically-caused condition, he/she not only has a duty to warn that person about the risk, but it is also important to inform the patient that the genetic test can generate information that might be relevant for others, especially for his/her family members. If the clinician judges that the results should be shared with family members, he/she may invite the patient to consider his/her responsibility towards their family. However, if the patient refuses to inform his/her family and if the counsellor cannot get his/her consent, it is possible that the counsellor will experience a problem of conscience in making a proportional weighing of the different values and duties at stake. The conditions that may justify a breach of confidentiality may be summarized as follows [7]:

(1) it is not possible to obtain a consent with respect to the disclosure of information;
(2) there is a concrete possibility that the lack of disclosure of the information could determine an appreciable damage, which would instead be avoided in the event of disclosure;
(3) the damage in case of non-disclosure would be very serious for very specific, identifiable individuals;
(4) all precautions will be taken so that the information provided relates exclusively to specific diagnoses or therapies;
(5) health-related damage has to be expected within the short term.

Nevertheless, genetic testing is not the only way we learn about such conditions, and physicians such as family doctors and geneticists are not the only healthcare professionals who acquire such knowledge or who face such situations [8].

At least in certain cases, forensic experts also acquire important medical knowledge and shoulder a similar burden. The management of IFs in medicine, for clinical or research reasons, raises ethical questions that are more difficult to address when forensic sciences are applied in real casework. In light of this difference, the role of the forensic expert in managing IFs relating to an identified or identifiable deceased person should be clarified, and his/her duty of confidentiality should also be evaluated.

What makes the present study original is that we will describe contemporary forensic situations in which IFs may occur, whether and to whom they may be disclosed, and some considerations regarding forensic experts concerning their moral commitment to warn relatives about IFs. Our descriptive analysis, which focuses on the forensic field, is different from the vast literature published on IF management in clinical and research fields.

The question of whether and how professionals should consider traditional values as guiding notions in the reporting of IFs in the context of forensic assessments is the focus of this article.

In this paper we will use the term “incidental findings” as the key phrase most commonly used to describe this situation.

## 2. Incidental Findings in the Forensic Setting

Today, in forensic settings, we can consider as incidental findings all the information that is neither related to the cause of death nor to the dynamic of the event or the scope of the forensic investigation. Specific forensic situations in which IFs can occur include, for example, molecular autopsy, the analysis of crime scene stains and human remains/cadaver identification. In general, in the context of forensic sciences, when IFs occur they may be of health-related or reproductive importance as well as perhaps revealing unexpected genetic relatedness. For example, unexpected discoveries during forensic examinations and analyses may reveal misattributed paternity/false beliefs about sibling relationships, identification of cancer or hereditary diseases not related to the cause of death, or the identification of a genetic mutation associated with a specific disease form. In any case, they may have an impact on the wellbeing of family members of the deceased person with whom the IFs are associated.

From a technical point of view, molecular autopsy employs genetic markers and predictive power of risk to assist in cause and manner-of-death investigations. The most frequent scenario includes genetic testing in negative autopsies, i.e., sudden unexpected deaths, when there are no findings from a standard autopsy [9]. Genetic testing can be part of a forensic post-mortem investigation and it can be used to confirm the presence of a disease-causing mutation. Recent progress in the fields of molecular biology and human genetics has enabled identification of the genetic etiology of many diseases. So molecular autopsy can lead to the revealing of genetic mutations associated with specific diseases, which may or may not be correlated with the cause of death. Obviously, if these mutations are related to the cause of death, then these findings cannot be considered to be incidental findings; whereas if they are unrelated to the cause of death they become relevant genetic information unrelated to the reason why forensic examination has been requested. Therefore, they can be considered, in the latter case, as incidental findings. For example, serious, genetically acquired diseases may emerge as an IF when other causes of death are identified. If an individual has an aggressive, hereditary form of cancer, knowing about the illness may prompt relatives to undergo testing and could lead to early detection and treatment [10].

Another example concerns the case in which DNA analyses are performed on presumed family members to identify a body or the remains of a body. In this case, any discrepancy revealed by the family relationship analysis would be immediately apparent to the investigators. At present, the genetic profiles obtained in forensic genetics investigations do not predict any disease-related characteristics of a person; otherwise, they may give information about the person’s sex (through the presence of X and Y chromosome markers) and about other visible physical traits (e.g., hair, eyes and skin color) [11,12]. However, the determination of sex, which is an integral part of the determination of the genetic profile, may reveal discrepancies between the chromosomal sex and the phenotype or social gender of the person concerned; some microdeletions identifiable with the haplotype of the Y chromosome could reveal fertility problems in the person concerned [13].

Furthermore, it may happen that in investigations aimed at establishing the identity of an unknown corpse or during paternity testing in deficiency cases (i.e., cases in which the mother is not tested, or the putative father is not tested and his genetic markers are deduced from those of relatives), it may be discovered that the tested reference subjects are not genetically related [14].

## 3. Discussion

How to handle this information raises ethical considerations for the forensic practitioner. He/she will have to consider many of the same questions already discussed in clinical practice and research: how many and which loci to sequence, what to report and whether ignoring sequence information is prudent. Certainly, there will be a number of ethical and legislative considerations in introducing new technologies in forensic genetics [15].

There are many differences between clinical and forensic IFs, mainly related to the involvement of the interested person, the type of professionals involved and the setting in which they operate.

### 3.1. New Technologies

The development of NGS (Next Generation Sequencing) techniques began in the early 2000s and revolutionized genomic research; although different approaches can be used, the basic principle is always that of sequencing DNA or RNA to obtain a very large amount of information. This type of analysis, used in the forensic field where it is often useful to have as much information as possible, can be a new source of IFs [16]. Massive parallel sequencing techniques are widely used in research and diagnostics and, if considered within forensic genetics, could replace the classic techniques of capillary electrophoresis for STR (Short Tandem Repeats) typing. It is likely that progress in DNA analysis and the determination of the genetic profile (the process of determining an individual’s unique DNA characteristics) will increasingly deliver the capacity to derive disease propensity as well as other kinds of medically relevant information from crime-scene stains or from biological samples collected from an individual for forensic purposes.

The possibility of identifying unexpected genetic information, more or less medically actionable, during post-mortem analyses with these new technologies will be increasingly concrete and more widespread in forensic daily practice. Therefore, in the near future, it will be increasingly important not only to understand what information may be collected through these techniques but also how to manage this information in the forensic field.

Even if present forensic analysis of crime scene stains could yield health-related information about the donor, it is still unclear what purpose would be served by such information.

A complicating fact is that the traditional limits of forensic genetic applications are changing with the increasingly widespread advent of new technologies. In fact, today, DNA genotyping in forensic medicine focuses on portions of DNA that are susceptible to mutations but appear to have no coding role in regulating or controlling specific cellular functions. What is defined as DNA phenotyping concerns the analysis of portions of the DNA that control certain visible characteristics. [17]. The progress of research in this direction could, in the near future, lead to the discovery of new IFs that are not predictable today [18].

When the forensic expert discovers IFs that are irrelevant for the purposes of his/her assessment but may be very important for other reasons, he/she is in a situation very similar to that of the clinician: is there a duty to inform the relatives of the deceased about important risks to their health? If this exists, how can it be balanced with the duty of secrecy towards the judge and with the duty of independence and impartiality in the preparation of the expert’s report? [19,20,21].

### 3.2. The Relationship between the Forensic Expert and the Judge

The medico-legal expert’s report, including diagnostic results and accompanying information, is submitted to the judicial system instead of the health care system and the judge is the only one to whom the forensic expert has to refer [12]. In case of the discovery of IFs during a forensic investigation ordered by the court, the problem that arises in some countries for the forensic expert is, first of all, whether and to whom these findings may be disclosed.

Considering the general definition of IFs, in forensics they are of no concern to the questions posed by the judicial authorities, they are of no concern to the cause of death and they have no relevance with respect to the reconstruction of the dynamics of the facts. Despite this, some information obtained in the course of investigations for forensic purposes could determine health and social risks for the relatives of the deceased, also on the basis of different family and socio-economic circumstances. Therefore, including IFs in the forensic expert’s report to the judge may be the only way to inform someone who is in charge of receiving technical information about the case. Furthermore, as has been found in other contexts, not only the degree of risk but also the magnitude of possible benefit from such health-related IFs depends on factors that investigators cannot be expected to anticipate [22]. The forensic expert is not authorized to have a direct relationship with the family and this means that he/she cannot inform relatives independently. On the other hand, family members often communicate only with the police or with the investigating magistrate. The forensic expert, however, depending on local law, may be authorized to communicate with the family with permission from the judge. However, when such approval is granted, forensic science practitioners will find themselves in possession of information regarding IFs that are unknown to relatives, and which the victim’s relatives may or may not want to know. When considering the management of such information it should be borne in mind that the forensic expert does not know what family members already know about the specific incidental finding. Moreover, as happens in other contexts, sometimes the positive and negative effects of the disclosure of some IFs can neither be predicted nor prevented by the investigators. In these cases, it might be useful to involve a clinical multidisciplinary team to investigate more deeply the clinical meaning of the IFs or to contact the family’s general practitioner. This seems to be impossible for forensic experts because it would be outside of the forensic objective, because the forensic expert has to respect “judicial confidentiality” and to report results or information only to the judge and is not authorized to use the resources of the NHS for justice purposes.

### 3.3. Permission to Contact Relatives

It must be said that the discovery of IFs in the forensic field implies a reflection on many of the ethical principles and issues that have already been discussed in the literature in the fields of clinical and biomedical research, and forensic experts are often forced to confront these issues [23,24,25].

One of the main issues concerns the relationship with the relatives of the deceased. In general, it is believed that physicians may be allowed to disclose information about a patient to relatives when the information concerns certain actionable genetic risks [26,27,28], while, in the forensic field, one must ask whether the coroner should respect the same moral constraints [29,30].

However, while in the clinical context permission to contact family members must be given by the patient, in the forensic field, if the incidental finding is related to a deceased subject, the moral obligation to have the permission of the interested party in order to reveal the information fails, but the legal and formal obligation for this to be authorized by the judge remains. In this context, the persons to whom the information refers cannot express their opinion on whether or not to communicate the IFs to their family members.

As previously stated, justification for the health professional to breach confidentiality depends on several conditions: the severity of the risk related to the seriousness of the possible outcome of IFs, the probability and the timing of the risk discovery, and the possibility that the knowledge of the risks can lead to prevention or to concrete benefit.

However, in forensics, the moral commitment to warn relatives should be counterbalanced with the duty to preserve the expert’s duty of confidentiality, and should be done only if certain conditions are fulfilled: one of these is that there is a high probability that harm will occur if the information is disclosed, or that the harm that identifiable individuals would suffer is serious. In other words, a forensic expert’s authorization to warn relatives could be justified when it is meaningfully actionable.

The characteristic that has traditionally been considered relevant in determining whether or not an IF should be revealed in the clinical setting s significance for the patient’s health in terms of level of penetration and curability. In the forensic field, the impact of clinical significance for the interested subject does not exist, as the subject is dead. From the previous discussion, we argue that the forensic expert’s duty to warn about IFs might be limited to informing the judge; otherwise, the forensic expert requires that judicial authorities grant permission to contact relatives directly and to share only as much information as is necessary to meet the meaningfully actionable Ifs, and only to those people who could benefit from information, or to contact and involve other healthcare professionals—for example, treating physicians—in sharing or delegating the disclosure of the unexpected medical information.

## 4. Conclusions

The potential for incidental findings in forensics should be considered in relation to the concrete benefit that can be expected from their disclosure and in relation to a new way of interpreting the duty of confidentiality of the forensic expert as a healthcare professional.

Even if this article outlines an ethical approach to the management of IFs in forensic investigations and analysis, further empirical and legal research is needed in order to close the current knowledge gaps.

Since national legislation for reporting of incidental findings in forensics is lacking, international or, at least, some local practice guidelines concerning medico-legal and forensic post-mortem genetic diagnostics need to be established since IFs could be used effectively for the benefit of individuals, families and society.

To give proper value to the potentiality of IFs in forensics, an internationally agreed set of recommendations are needed on how to make proper use of the samples and how to report the results in a standardized way.

**Figure 1:**
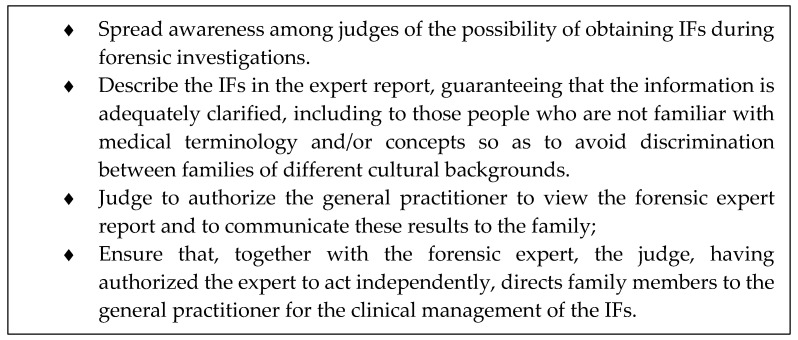
The box contains some indications of best practice for the management of incidental findings in forensics.

Because of the challenges related to the management of IFs in forensics, some elements of good practice can be suggested (See Figure 1).

Good governance and robust ethical discussion of the topic can anticipate and respond to these new challenges; in fact, it governance is not merely a means to impose sanctions when things go wrong, but can promote uniformity of behavior in different jurisdictions and enable forensic experts to act in such a way as to discharge their duties towards all the parties involved.
